# The Use of a Barley-Based Well to Define Cationic Betaglucan to Study Mammalian Cell Toxicity Associated with Interactions with Biological Structures

**DOI:** 10.3390/pharmaceutics15072009

**Published:** 2023-07-23

**Authors:** Malgorzata Tymecka, Katarzyna Hac-Wydro, Magdalena Obloza, Piotr Bonarek, Kamil Kaminski

**Affiliations:** 1 Doctoral School of Exact and Natural Sciences, Faculty of Chemistry, Jagiellonian University, Gronostajowa 2, 30-387 Kraków, Poland; m.tymecka@doctoral.uj.edu.pl; 2 Faculty of Chemistry, Jagiellonian University, Gronostajowa 2, 30-387 Kraków, Poland; katarzyna.hac-wydro@uj.edu.pl (K.H.-W.); m.obloza@uj.edu.pl (M.O.); 3 Department of Physical Biochemistry, Faculty of Biochemistry, Biophysics and Biotechnology, Jagiellonian University, Gronostajowa 7, 30-387 Krakow, Poland; piotr.bonarek@uj.edu.pl

**Keywords:** polycations, betaglucans, toxicity

## Abstract

Among potential macromolecule-based pharmaceuticals, polycations seem particularly interesting due to their proven antimicrobial properties and use as vectors in gene therapy. This makes an understanding of the mechanisms of these molecules’ interaction with living structures important, so the goal of this paper was to propose and carry out experiments that will allow us to characterize these phenomena. Of particular importance is the question of toxicity of such structures to mammalian cells and, in the work presented here, two lines, normal fibroblasts 3T3-L1 and A549 lung cancer, were used to determine this. In this work, three well-defined cationic derivatives of barley-derived betaglucans obtained in a reaction with glycidyltrimethylammonium chloride (BBGGTMAC) with different degrees of cationization (50, 70, and 100% per one glucose unit) and electrostatic charge were studied. The studies address interactions of these polymers with proteins (bovine serum proteins and BSA), nucleic acids (DNA), glycosaminoglycans (heparin), and biological membranes. The results described in this study make it possible to indicate that toxicity is most strongly influenced by interactions with biological membranes and is closely related to the electrostatic charge of the macromolecule. The presentation of this observation was the goal of this publication. This paper also shows, using fluorescently labeled variants of polymers, the penetration and impact on cell structure (only for the polymer with the highest substitution binding to cell membranes is observed) by using confocal and SEM (for the polymer with the highest degree of substitution, and the appearance of additional structures on the surface of the cell membrane is observed). The labeled polymers are also tools used together with dynamic light scattering and calorimetric titration to study their interaction with other biopolymers. As for the interactions with biological membranes, lipid Langmuir monolayers as model membrane systems were used.

## 1. Introduction

The development and refinement of polymer synthesis procedures have changed human life more than any other discovery in the field of chemistry [1]. These disruptive technologies have entered pharmaceutical science in the 21st century as well [2], and as it seems before our eyes, they could change it forever. The greatest potential is in systems where biological activity results directly from the physicochemical properties of macromolecules. Particularly interesting in this respect are polyelectrolytes that, thanks to the presence of a well-defined charge, not only show high solubility under physiological conditions but also susceptibility to interactions mainly of an electrostatic nature, but not exclusively. This can include interactions between oppositely charged polyelectrolytes, proteins, and lipid membrane structures. Among polymers possessing a well-defined charge, perhaps the most extensively studied structures today are polycations, which, it is postulated, can significantly interact with mostly negatively charged biological systems at the molecular or cellular level. Studying these interactions and correlating them with toxicity on cellular models of well-defined polycations is the subject of this publication.

Polycations due to electrostatic attraction interact with negatively charged biopolymers present in living organisms, mainly nucleic acids and glycosaminoglycans. The properties of these biologically active polyanions change as a result of such interactions and usually lead to the formation of particles with diameters on the submicrometer or micrometer scale [3]. These processes can be directly used in medical and biological applications to stabilize nucleic acids in gene therapy and transfection [4] or neutralize the anticoagulant activity of heparin [5], which is a glycosaminoglycan [6]. In the literature, one can also find reports of practical medical use of polycations in applications related to interactions with proteins, as tools for their delivery and stabilization (insulin [7]) or stimulation or inhibition of their action (growth factor [8]).

Another noteworthy biological property of polycations is their biocidal properties, in particular antibacterials [9]. Recent years have also brought reports of antifungal properties [10] confirmed on in vivo infection models [11]. In the era of the growing problem with drug resistance of pathogenic bacteria and fungi [12], polycations could potentially be the foundation for a new family of antibiotics. The problem is the issue of the toxicity of these structures toward cells of higher organisms, in particular mammalians. For polycations to be used as biologically active compounds and drugs, it is necessary to reduce their toxicity or create selectively toxic polycations.

To achieve this goal, the phenomena responsible for the toxicity of polycations toward mammalian cells have to be indicated. In the literature, one can find relatively few works [13,14,15] postulating such a mechanism, which are very often mutually exclusive. This can be explained by the complexity of the processes, but it would be particularly valuable, if possible, to identify one universal process. The key to understanding the mechanisms of the toxicity of polycations is the ability to correlate the physicochemical parameters of polymers and their effects on cell welfare. Particularly important in this regard is the molecular weight of polymers and the distribution of positive charges along the main chain. This raises the question of what chemical structures to use for such studies. We propose to use polymers of natural origin and modify them to obtain a series of similar polymers varying only in positive charge distribution along the main chain.

For this type of research, betaglucans seem to be particularly useful. They manifest high resistance to chemical depolymerization in a wide range of pH in aqueous solutions [16] and are, at the same time, susceptible to reactions with epoxides, which allows for the easy introduction of cationic amine groups [17]. Such reactions can be performed using glycidyltrimethylammonium chloride, GTMAC, and are allowed to obtain many polycations described in the literature based on chitosan [18], dextran [19], or cyclodextrins [20]. This makes it possible to design polycations with very well-defined properties in terms of the degree of cationic modification and molecular weight. 

Betaglucans (BGs) are polysaccharides, in which glucose molecules are organized in a linear chain. They are typically classified based on their molecular structure, with the most prevalent types being beta-1,3-glucans, beta-1,4-glucans, and mixed-linkage beta-glucans [21]. BGs are often found in nutritional supplements, functional foods [22], and biomedical applications [23], and may be derived from a variety of natural sources including mushrooms, oats, barley, and yeast [24]. Numerous studies have investigated how BGs affect the immune system, including how they can activate immune cells, increase immunity to infections, and control inflammatory reactions [25,26]. Several preclinical and clinical studies investigated the use of betaglucans as an adjuvant therapy due to their potential role in the treatment of cancer. For instance, the use of BG as an immunomodulatory drug in cancer therapy [27].

## 2. Materials and Methods

### 2.1. Materials

Wholemeal barley flour, which originated at the Gospodarstwo Rolne Mateusz Gren Radcze Farm in Poland, was used. Ethanol (96%, p.a.), HCl (36%, p.a.), NaCl (p.a.), Na_2_SO_4_ (p.a.), and acetic acid (99%) were bought from Chempur (Piekary Slaskie, Poland). Glycidyltrimethylammonium chloride (GTMAC, 50% in water) was bought from Sigma-Aldrich (Burlington, MA, USA). Dialysis membrane Spectra/Por MWCO 3500 Cal Roth (Karlsruhe, Germany) was used. For the GPC/SEC measurements for all polymers, the column set of PolySep-SEC GFC-P 2000, 4000, and 6000 LC Column 300 × 7.8 mm (Phenomenex, Torrance, CA, USA) was used. For cell culture, 3T3-L1 (ATCC CL-173) and A549 (ATCC CRM-CCL-185) cells purchased from American Type Culture Collection (ATCC) (Manassas, VA, USA) were used. Cell medium (DMEM) and additives were purchased from Sigma-Aldrich (Burlington, MA, USA). Gibco Ham’s F-12K (Kaighn’s) cell medium was purchased from ThermoFisher Scientific (Waltham, MA, USA).

For the lipid monolayer studies, the high purity (>99%) phospholipids: 1-palmitoyl-2-oleoyl-sn-glycero-3-phosphocholine (POPC), egg yolk sphingomyelin (SM) (purchased from Avanti Polar Lipids Inc), and cholesterol (Chol) (supplied by Sigma-Aldrich) were used. The 1/1/1 mixed POPC/SM/Chol monolayer was treated as a model erythrocyte membrane. The stock solutions of POPC and SM were prepared by dissolving the lipids in a chloroform/methanol 9:1 *v*/*v* mixture (HPLC grade, ≥99.9%, Sigma-Aldrich), while cholesterol was dissolved in chloroform. The components of phosphate-buffered saline (PBS), sodium chloride, potassium chloride, disodium hydrogen phosphate, and potassium dihydrogen phosphate were high-purity compounds (>99%) supplied by POCH S.A. In all experiments, ultrapure water (Milli-Q, resistivity ≥ 18.2 MΩ∙cm) was used.

### 2.2. Polymer Synthesis

#### 2.2.1. Isolation of Betaglucan

Betaglucan was isolated from whole-grain barley flour according to the recipe described earlier [10,28]. Briefly, the procedure went as follows. A total of 20 g of barley flour was suspended in 200 mL of water. The pH of the suspension was adjusted to 7.6 using a 10% potassium carbonate solution, and the mixture was then heated for 30 min at 45 °C and centrifuged for 30 min at 4 °C at 4940 G. The obtained supernatant was brought to pH 4.5 with 2 M HCl and centrifuged for 30 min at 4940 G in 4 °C. The betaglucan present in the supernatant was precipitated with ethanol (volume ratio 1:1 supernatant: ethanol) and left overnight at 8 °C. The mixture was then centrifuged for 10 min at 3780 G, and the resulting precipitate was dried under reduced pressure at 40 °C. The reaction yield was 3%.

#### 2.2.2. Cationization of Betaglucans with Varying Degrees of Substitution

Cationization of the isolated crude betaglucan was carried out as described in our earlier publication [10]. Briefly, for BBGGTMAC1 and BBGGTMAC2 polymers, crude betaglucan was suspended in 50 cm^3^ of water, followed by the addition of 200 mg of NaOH and GTMAC (exact amounts according to the Table 1). The reaction was carried out at 60 °C for 4 h, and then the solution was transferred to a dialysis tube and dialyzed against water for 4 days, changing to fresh water once a day. The resulting mixture was centrifuged for 5 min at 10,000 rpm, and the supernatant was lyophilized. In the case of BBGGTMAC3, the already pre-modified BBGGTMAC2 was used as a substrate, and the rest of the steps were performed analogously as described above using 200 mg NaOH and 6 mL GTMAC. Reaction time, temperature, purification, and isolation were realized analogously. The yield of the cationization process was 80%

#### 2.2.3. Fluorescent Labeling of Polycations

In order to be able to use fluorescence spectroscopy to study the interaction of polycations with biological structures and indicate the sites of accumulation in cells, the three obtained polycations were fluorescently labeled with rhodamine B. The synthesis reactions were realized as follows. A total of 250 mg of each polymer was dissolved in a mixture of 10 mL DMSO and 1 mL water, and then 2 drops of pyridine and 10 mg of rhodamine B isothiocyanate were added and heated to 100 °C, stirring all the time. Reactions were carried out for 4 h and then dialyzed for 5 days, changing water every 1 day. The product was isolated from the solution using freeze drying. The yield of the process was 70%. The amounts of the attached fluorophore particles were determined by UV-VIS measurements of polymer solutions (of known concentration), and the corresponding standard curves were prepared for solutions of rhodamine isothiocyanate B alone. Polymers were labeled with 2–6% of rhodamine per glucose unit.

#### 2.2.4. Depolymerization Process of BBGGTMAC2

A total of 12 mL of 1% BBGGTMAC2 solution was introduced into the Ubbelohde capillary viscometer (inner diameter of the capillary 1.2 mm). The flow time of the solution was measured after the addition of 5 mg of betaglucanase. After 45 min, another portion of 25 mg of betaglucanase was added, and the measurements continued for another 45 min.

#### 2.2.5. Confirmation of Obtaining the Intended Chemical Structure

**Figure 1:**
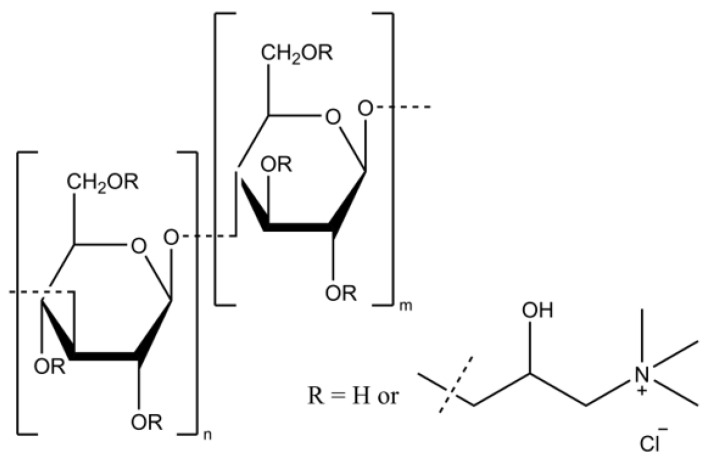
The structure of the obtained polycations.

The effective isolation of betaglucans, cationization, and the achievement of the intended chemical structures (shown in Figure 1) were confirmed using ATR FTIR and ^1^HNMR spectroscopy (Supplementary Materials Figures S1 and S3). The degree of substitution with the cationic group to the betaglucan molecules was determined by ^1^H NMR spectra and combustion elemental analysis. The molecular masses were determined by the GPC measurements.

### 2.3. Impact of Polymers on Cell Line Models

Embryo mouse fibroblast 3T3-L1 and adenocarcinomica human alveolar basal epithelial cells A549 were used to assess the toxicity of the polymers. For the cell line 3T3-L1, Dulbecco’s modified Eagle’s medium supplemented with fetal bovine serum for a final concentration of 10% (*v*/*v*), and 1% (*v*/*v*) penicillin–streptomycin solution was used. For the cell line A549, Gibco Ham’s F-12K (Kaighn’s) medium was used. Cultures were incubated at 37 °C in an atmosphere containing 5% carbon dioxide. For each assessment, the cells were seeded at 6 × 10^4^ per well into 24-well plates and grown for 24 h. Then, the medium was changed to full medium (containing 10% fetal bovine serum) or serum-free medium, and a polycation solution (in serum-free media) was added and let to grow for the next 24 h to assess cytotoxicity using the crystal violet assay [29]. Three independent measurements were made for each concentration of the polymers, and the results presented are as the arithmetic mean ± standard deviation (SD).

### 2.4. Lipid Monolayer Studies

The surface pressure (π)/area (A) isotherms were recorded on a KSV-NIMA (model KN2002) double-barrier Langmuir–Blodgett trough in a total area of 275 cm^2^. The subphase PBS buffer or polymer solutions were used. The concentrations of the polymers in the subphase were 1, 5, or 10 μg/mL. The monolayer was formed by deposition of the solution of lipids onto the subphase with a Hamilton microsyringe (±2.0 μL). After evaporation of the solvent, the monolayer was compressed by the movable barriers at a constant rate of 10 cm^2^/min. The changes in the surface pressure during film compression were automatically recorded (±0.1 mN/m) by a surface pressure sensor equipped with a Wilhelmy plate made of chromatographic paper (ashless Whatman Chr1). The experiments were carried out at 20 °C, and the temperature was controlled by a Julabo thermostat. The measurements were repeated to ensure consistent results (the error for the area per molecule did not exceed 0.2 A^2^/molecule). To compare the ability of the studied polymers to penetrate the lipid monolayer, the polymer solutions were injected into the film and compressed to a desirable surface pressure. Thus, the mixed film formed on PBS was compressed to the target surface pressure. Then, it was left for equilibration to the initial surface pressure π_in_ (5 or 30 mN/m), and the polymer solution was injected into the subphase to a final concentration of 10 μg/mL. During the experiments, the subphase was continuously stirred, and the changes in the surface pressure π in time were recorded. These experiments were repeated to obtain results that did not differ by more than 2%.

### 2.5. Biomacromolecule Polycation Interaction Studies and Zeta Potential Measurements

The interaction of polycations with proteins was investigated by measuring the dynamic light scattering in a mixture of serum or bovine serum albumin (with a concentration of 1 mg/mL) and polymers (in the concentration range of 0–500 µg/mL). The interaction of polycations with DNA (with a DNA concentration of 1 mg/mL) and polymers (in a concentration range of 0–2 mg/mL) was investigated accordingly. Results for DNA are complemented by fluorescence measurements for the fluorescently labeled polymer variant (0.1 mg/mL concentration in 0.1 M NaCl) in mixtures with various concentrations of albumin (in the 0–500 µg/mL concentration range). Zetasizer Nano ZS and fluorescence spectrophotometer Hitachi F-7000 instruments were used for DLS and fluorescence measurements, respectively. The same apparatai were used for zeta potential measurements for 1 mg/mL concentrated polymer solutions in water. Data on the interaction of polycations with DNA and albumin were supplemented by Isothermal Titration Calorimetry (ITC) measurements. ITC measurements were performed using a VP-ITC instrument (MicroCal, Northampton, MA, USA). All experiments were conducted in PBS at 37 °C. All solutions were degassed for 5 min under a vacuum before the experiments. For albumin, typically 10 μL aliquots of the 200 µM polymer solution were added by 30 injections into a calorimeter cell with a volume of 1435.5 μL filled with a 20 µM BSA solution or only PBS (reference measurements). The injection rate was 0.5 μL/s, and the interval between injections was 4 min. For DNA, typically 10 μL aliquots of the 5 mM bp DNA solution were added by 30 injections into a calorimeter cell with a volume of 1435.5 μL filled with 500 µM (as the concentration of monomer) of polymers or only PBS (reference measurements). The injection rate was 0.5 μL/s, and the interval between injections was 3.5 min. To ensure proper mixing after each injection, a constant stirring speed of 300 rpm was maintained throughout the experiment. Data analysis was performed globally for two titrations using a single binding site interaction model with the stoichiometry parameter, association constant, enthalpy, and offset, with a shared K_app_ value, using MicroCal Origin scientific plotting software.

The interaction of polycations with glycosaminoglycans (GAGs) was studied using the model GAG heparin and a method based on studying the decrease in the concentration of this polyanion in the presence of polycations using azure A dye as described in the literature [3]. On the practical side, the measurement involves preparing appropriate mixtures of polycations (varying concentrations ranging from 0 to 960 µg/mL) and heparin (concentration 50 j.m/mL) centrifugation (centrifugation time 5 min and centrifugation speed 2500 per minute) and measuring the absorbance of the 1:1 mixture of the supernatant and dye solution (dye concentration 8 × 10^−5^ M). The decrease in absorbance for 630 nm is proportional to the decrease in the concentration of heparin.

### 2.6. Microscopic Imaging of Cells

3T3-L1 cells were seeded on coverslips and after 24 h, fluorescently labeled polymers (for confocal) or ones that were not labeled (for SEM) were added all at concentrations of 100 μg/mL. Culture conditions were the same as in the experiments to evaluate the effect on proliferation (serum medium described above). After another 24 h, the slide was washed in warm PBS and fixed (10 min at room temperature) with 4% buffered formalin (for confocal) or glutharaldehyde in PBS (for SEM). For the confocal microscopy experiment, cells were washed thrice with PBS, and nuclei (DNA) were stained with DAPI (0.1 μg/mL) for 10 min at room temperature. Cells were then washed with PBS 3 times, and coverslips with immunostained cells were mounted on glass slides and sealed. Fluorescent images were acquired with an A1-Si Nikon (Tokyo, Japan) confocal laser scanning system built onto a Nikon-inverted microscope Ti-E using a Plan Apo 100×/1.4 Oil DIC objective. Images were recorded at a resolution of 1024 × 1024, while dyes were excited with 405 and 532 nm diode lasers, respectively. Three-dimensional fluorescence images were constructed using NIS-Elements AR 3.2 software. In the case of the SEM experiment, after the sample is fixed, it is transferred to a 70% ethanol solution for another 10 min. This action is repeated for successively increasing concentrations of ethanol: 80, 90, and 100% (dry ethanol). The final step is to transfer the slide to the hexamethyldisilazane (HMDS) for 5 min and, after removal and air-drying for 10 min, the sample was sputtered with a 5 nm layer of gold. The samples obtained were imaged using an environmental scanning electron microscope SEM Phenom-World PRO (Pik Instruments, Piaseczno, Poland). These procedures are also described in our earlier publications [30].

## 3. Results

### 3.1. Basic Physical and Chemical Characteristics of the Polymers

The procedures described in this work for the isolation of betaglucan and purification from cereals are based on earlier descriptions in the literature [10] where effective isolation of these polysaccharides has been confirmed. The cationization reaction is an expansion of a procedure we also described in an earlier paper [21]. Changes in the procedure made it possible to obtain polycations with varying degrees of cationic groups, and the degree of modification was determined by two independent methods on the basis of elemental analysis and ^1^H NMR spectra. Compared to the earlier work describing these reactions, we used different and multiplied methods to determine the degree of modification, which we postulate gives more reliable values. Also improved was the system used to determine the molecular weights, whereas in the case of this work, the GPC was also used with a three-column system, which improved the applicability of the method and its resolution, allowing us to obtain more reliable results. The molecular weight distribution of the obtained polycations is relatively low for derivatives of macromolecules of natural origin. Despite the differences in the composition of the reaction mixtures and the repetition of the synthesis in the case of polymer BBGGTMAC3, the differences between the mass dispersion between the polymers are very minor and should not affect the biological properties.

For the three polymers obtained, the desired polysaccharide structures with attached GTMAC molecules were obtained, which was confirmed by performing an ATR FTIR and ^1^H NMR spectra of these compounds. The full spectra with descriptions of characteristic peaks can be found in the Supplementary Materials. Above is a table containing other important parameters characterizing the polymers, such as the degree of substitution with the cationic group, molecular weight, and zeta potential. The zeta potential increases with the degree of cationic modification, which confirms that the more GTMAC groups are attached, the greater the positive charge of the macromolecule. The values obtained from the elemental analysis may be considered to be partly overestimated due to the presence of a small amount of nitrogen in the crude purified betaglucan (details in Supplementary Table S1).

The susceptibility of the obtained polymers to biodegradation was confirmed by the demonstration of the possibility of depolymerization of these polycations using betaglucanase, which in nature degrades crude betaglucans. The depolimerisation process was conducted for BBGGTMAC2. With the addition of betaglucanase, which is known to break down polysaccharides through the process of hydrolysis, the viscosimeter flow time of the polication solution decreased [31], which means a decrease in viscosity, and thus the molecular weight of the macromolecules is present in the solution. It is an initial confirmation of the ability of the polycation to undergo biodegradability (details in Supplementary Table S3).

Since the effect of the hydrophobicity of macromolecules has a significant impact on the bioactivity and toxicity of a given compound, logP values were calculated for structures that in a simplified way approximated the obtained polymers (details of the choice of the structures and method of counting are in Table 2). The data obtained relatively large negative values and reflect the hydrophilic nature of the polycations of macromolecules having electrostatic charge. The logP values change slightly with BBGGTMAC1 and BBGTMAC2, increasing with the cationization degree. Between BBGGTMAC2 and BBGGTMAC3, there is no significant change in the logP value.

### 3.2. Impact of Polymers on Toxicity and Proliferation

**Figure 2:**
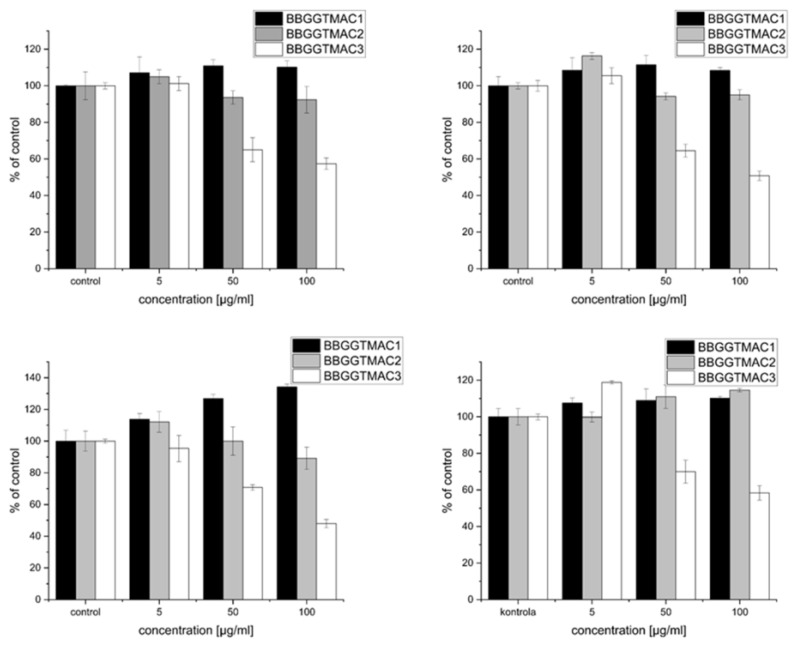
The effect of polycations on cell survival: top—A549 cells on the left without serum and on the right with serum, down—3T3 cells on the left without serum and on the right with serum.

The work presented here shows the effect of three polycations differing in the degree of cationic modification on two cell lines: the lung cancer cell line A549 and normal mouse fetal fibroblasts 3T3-L1 (results in Figure 2 below). In the case of the polymers tested, the effect on cancer cells, as well as normal cells, strongly depended on the degree of modification. A significant negative effect occurs for polymer BBGGTMAC3 under serum (negative effect on proliferation) and serum-free (toxicity) conditions. For both lines and under both conditions of the experiment, for a concentration of 50 μg/mL, the number of cells drops to about 60% of the control, while for a concentration of 100 μg/mL, it drops below 50%. For the other two polymers, there is no significant decrease and in the case of fibroblasts; even a pro-proliferative effect was observed for systems with serum.

An attempt to explain why this phenomenon occurs is the purpose of this publication. Particularly important here is the difference in properties between polymers BBGGTMAC2 and BBGGTMAC3, whose molecular weights are very similar, and the degree of modification varies by about 30%, which makes it possible to correlate these phenomena directly with the degree of modification. The research hypothesis is that the negative effect on cells is the result of interactions with one or more critical structures present in cells and their environment (bilayer, proteins, nucleic acids, glycosaminoglycans), which will vary quantitatively or qualitatively for these three polymer structures.

### 3.3. Interaction of Polycations with Proteins

**Figure 3:**
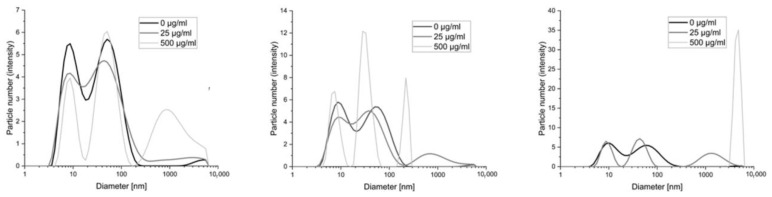
The diameters of the objects formed based on dynamic light scattering as a result of the interaction of polymers with bovine serum albumin. From left to right: BBGGTMAC1, BBGGTMAC2, BBGGTMAC3.

In this work, methods based on imaging the reaction products of polycations with serum and albumin measured by dynamic light scattering or fluorescence (fluorescently labeled polycations) were used as a model for the interaction of these polymers with proteins. Images of the change in diameter of the objects present in the serum–polymer mixtures are in the figure below (Figure 3).

The smallest changes occur with polymer BBGGTMAC1, where only the highest concentration (500 μg/mL) results in the appearance of objects that can be considered a product of the reaction with serum proteins. For the other two polymers with a higher degree of cationization already for lower concentration (25 μg/mL), we observed the formation of such structures. In their case, the highest concentrations, in particular for polymer BBGGTMAC3, manifest large aggregates of protein polycation systems.

**Figure 4:**
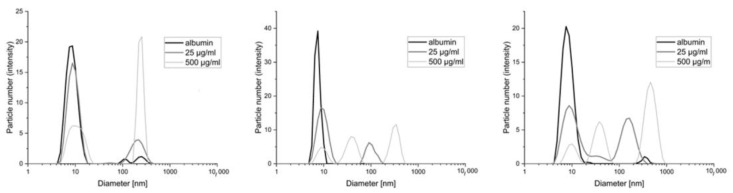
The diameters of the objects formed based on dynamic light scattering as a result of the interaction of polymers with human albumin. From left to right: BBGGTMAC1, BBGGTMAC2, BBGGTMAC3.

The image of the distribution of the particles in the mixture of albumin polycations includes a population of 10 nm originating from albumin and two more originating from the polymer. The first smaller one coincides with the location of those derived from albumin, while the second one, larger in diameter but smaller in terms of particle count (no more than 20% in all cases), falls in the few hundred nm and comes from polymer aggregates. The presence of aggregates, in this case, may come from the incomplete solubility of the obtained polycations, which is associated with large molecular weights and the non-homogeneous nature of the reaction substrate (which is to be expected for a natural polymer). We observed a lack of distinct structures that could come from the products of the polymer albumin reaction. This means that by comparing polymers with various degrees of modification, we see that the difference between the polymers is not noticeable in the case of the results for albumin alone (Figure 4). It is impossible to indicate the polymer with the strongest interaction. Additionally, some of the objects overlap with the aggregates found in pure polymer solutions (data in Supplementary Figure S6). Therefore, additional measurements were performed using ITC.

The heat effects obtained during BSA titration with polycations after subtracting the heat of polycation dilution are shown in Supplementary Figure S4. There are no significant changes in the measured heat values during the titration (BBGGTMAC1 and 2), or linear dependence occurs (BBGGTMAC3). Such a result indicates no interaction or very low affinity between the polycations and BSA. The non-zero signal, despite considering polycation dilution heats, is probably due to differences in the solvent composition.

### 3.4. Interaction of Polycations with DNA and Glycosaminoglycans

Glycosamine glycans are one of the most important non-protein biopolymers present in the intercellular matrix. We are talking about chondroitin, heparan sulfate dermatan, and heparin, which are found in all tissues and organs; so, it makes sense to study how the three polymers obtained interact with them. Heparin, the best-known and most medically used glycosaminoglycan [3], was used here as a model GAG. The decrease in heparin concentration influenced by polycations confirms our earlier observations described in the literature [3]. It indicates the interaction of these compounds with GAGs, and the values of the amount of polycation necessary to bind 1 mg of heparin can be taken as a measure of this interaction. The data obtained sequentially for polymers BBGGTMAC1, BBGGTMAC2, and BBGGTMAC3 (0.87; 0.82; 0.65) show that the binding efficiency increases with the degree of cationization, but these changes are moderate (up to 20% between polymers). The analogy in the heparin concentration drop curves (Supplementary Figure S2) indicates that these are only quantitative changes.

Analyzing the results of the changes in polymer fluorescence (full correlations showing the change in fluorescence intensity in Supplementary Figure S5) in the presence of increasing concentrations of DNA, one can observe that the change precedes first rapidly for concentrations of 25 to 125 µg/mL, and then changes are less significant for concentrations of 150–250 µg/mL. This is a characteristic relationship for all three polymers, and the course of the curves for DNA-induced polycation fluorescence change resembles the curves of heparin-binding by the polymers. These changes in fluorescence indicate an interaction between oppositely charged polycations and DNA, which is further confirmed by the formation of micrometric polyplex particles in these systems, as shown by DLS measurements (data in Supplementary Figures S7 and S8). These are objects in the size range from 0.5 to 6 micrometers whose diameters decrease with increasing polycation concentration. This effect is due to the higher DNA value of the zeta potential of polymers, which causes, for the whole polyplex, an increase in the value of this potential, which limits aggregation and consequently causes a decrease in the diameter of the particles. The pattern of fluorescence change (the slope of the curve and its form) and the polyplex particle size change for mixtures of polycations and DNA solutions, when we compare polymers with different degrees of substitution, does not show significant differences.

Due to the great importance of all interactions with nucleic acids in biological systems, the interactions of the polymers studied with DNA were additionally tested using ITC calorimetry (Supplementary Figure S8). Due to the polydispersity of the polymers and DNA, the analysis of the experiments was based on the concentration of the polymer monomers and the concentration of base pairs for DNA. No significant thermal effects were observed for BBGGTMAC1. In the case of BBGGTMAC2 and 3, the addition of DNA to the polymers was accompanied by exothermic thermal effects, which disappeared at about a molar ratio of 1:1. In addition, for these polymers, a pronounced opacity was observed in the reaction mixture, which was not apparent for measurements with BBGGTMAC1. According to the above observations, the interactions of the tested polycations with DNA are varied, not present for BBGGTMAC1, and significant for the others. To quantitatively describe the interactions, an analysis was performed based on the model of a single class of binding sites (continuous lines shown in Supplementary Figure S8). The resulting parameter values are shown in Supplementary Table S2. The formation of macroscopic aggregates during the interaction of the polymers with DNA indicates a non-equilibrium process; hence, the determined parameters should be interpreted with caution. Nevertheless, based on the values of equilibrium constants, it can be concluded that BBGGTMAC3 binds DNA with seven times higher affinity in comparison to BBGGTMAC2. The increase in affinity is likely due to the higher degree of cationization in BBGGTMAC3. Surprisingly, there is no apparent interaction of BBGGTMAC1 with DNA in ITC. Since such interaction was confirmed in fluorescence and DLS measurements, a possible explanation is the near-zero enthalpy of the process, resulting in the absence of significant thermal effects during titration. Although, on the basis of ITC, there is a difference in the interaction of the two most cationic polymers with DNA. It is minor, and the discrepancy in relation to the results obtained by investigating this process using DLS and fluorescence indicates that calorimetry results cannot be considered conclusive.

### 3.5. Interaction of Polycations with Biological Membranes

**Figure 5:**
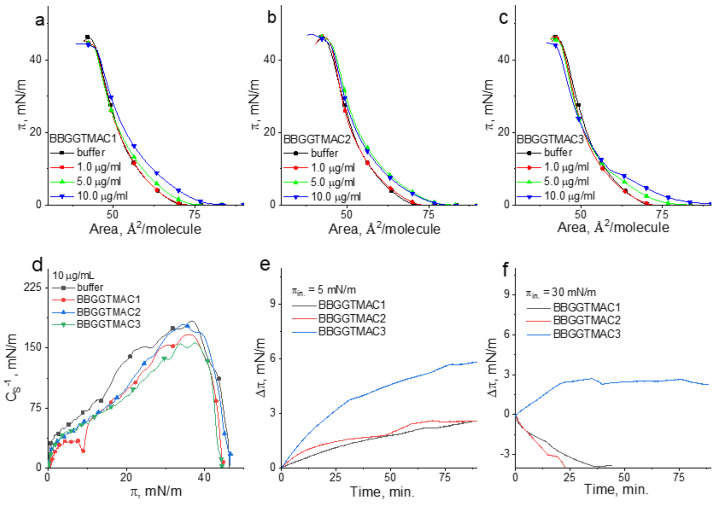
The surface pressure/area isotherms for the mixed monolayer on the buffer and polymer solutions (**a**–**c**); the compressional modulus vs. the surface pressure plots for the mixed monolayer on the buffer and polymer solutions (**d**); the results of the penetration experiments at 5 and 30 mN/m (**e**,**f**).

In Figure 5, the π/A isotherms for the POPC/SM/Chol monolayer on the buffer and polymer solutions are shown. From these curves, according to the equation below, the compressional modulus (*C_S_*^−1^) was calculated [32].

*C_S_*^−1^ = −*A* (*dπ*/*dA*) (1)

In the above equation, *A* is the mean area per molecule value at a given surface pressure *π*. This parameter can be interpreted as follows: the higher *C_S_*^−1^ values, the higher the condensation [32], and the lower the lateral elasticity of the model membrane [33]. The compressional modulus values were presented as the function of the surface pressure in Figure 5d. (The error of the calculated *C_S_*^−1^ values does not exceed 2%.) In the same figure (Figure 5e,f), the results of the penetration experiments at low and high surface pressure are shown.

As can be noticed under compression of the film on the buffer, the surface pressure increases monotonically from ca. 72 Å^2^/molecule to the collapse occurring at ca. 48 mN/m. Based on the maximal value of the compressional modulus, the state of this film can be classified as liquid condensed (LC). In the presence of polymers, the course of the curve is changed; however, all the collected results evidence the differences in the effect of BBGGTMAC1 vs. BBGGTMAC2 and BBGGTMAC3 and between BBGGTMAC2 and BBGGTMAC3 on the model lipid system.

Namely, in the presence of BBGGTMAC1 molecules, the isotherms shift to larger areas, and the compressional modulus values and the collapse surface pressure decrease. This means that the polymer decreases the condensation of the monolayer and makes it less stable. Since the values of ∆π (Figure 5e,f) are positive, both at low and high surface pressures, it can be concluded that BBGGTMAC1 molecules are able to incorporate into the lipid monolayer and accommodate it, even at the membrane-related surface pressures. However, it is important to note that, in fact, the influence of this polymer is pronounced only at the highest applied concentration. This is important when the toxicity of this polymer is considered.

The effect of the BBGGTMAC2 and BBGGTMAC3 molecules on the lipid monolayer is different. Namely, they are able to incorporate into the monolayer only at low surface pressure. Nevertheless, ∆π values at π = 5 mN/m (Figure 5e) are very similar for both polymers, and they are significantly lower than those obtained for BBGGTMAC1. Thus, the insertion of BBGGTMAC2 and BBGGTMAC3 to the model membrane is much weaker compared to BBGGTMAC1 molecules. Moreover, in the course of the isotherms taken on BBGGTMAC3 containing subphase, the deformation is well-noticed (it is reflected as the minimum in C_S_^−1^ vs. π plot). This deformation can be interpreted as the indicator of the exclusion of the polymer molecules from the monolayer. Interestingly, the curves recorded in the presence of BBGGTMAC2 do not shift to the larger areas in a systematic way; the isotherms for the two highest concentrations practically cover each other. Both results for BBGGTMAC2 and BBGGTMAC3 suggest that with the increasing polymer concentration or increasing surface pressure, the molecules cannot be incorporated into the model membrane. The negative values of ∆π at 30 mN/m obtained in the penetration experiments (Figure 5f) confirm the latter conclusion. However, the obtained results also evidence that both BBGGTMAC2 and BBGGTMAC3 are removed from the lipid environment together with the monolayer material, and in this manner, they alter the monolayer properties. This effect is especially pronounced for BBGGTMAC3 and also reflects also a well-noticed shift of the isotherms to the lower areas at higher surface pressures.

The results of the monolayer experiments can be linked to the bilayer system and analyzed from the point of view of the effect of the substance on the membrane only when the surface pressure region of 30–35 mN/m is considered [33]. The results obtained herein evidenced that at 30 mN/m, BBGGTMAC1 molecules incorporate into the lipid film; however, this effect is pronounced only at the highest concentration of molecules. The remaining polymers are not able to insert into the monolayer; however, they change their properties by the removal of the monolayer material from the interface. For BBGGTMAC3, this effect is well-pronounced, even at the lowest polymer concentrations. Therefore, it can be concluded that BBGGTMAC3 molecules may cause alterations at the level of the membrane more easily and according to a different mechanism than BBGGTMAC1.

### 3.6. Interaction of Polycations with the Whole Cell Structure—Confocal Microscopy and SEM

**Figure 6:**
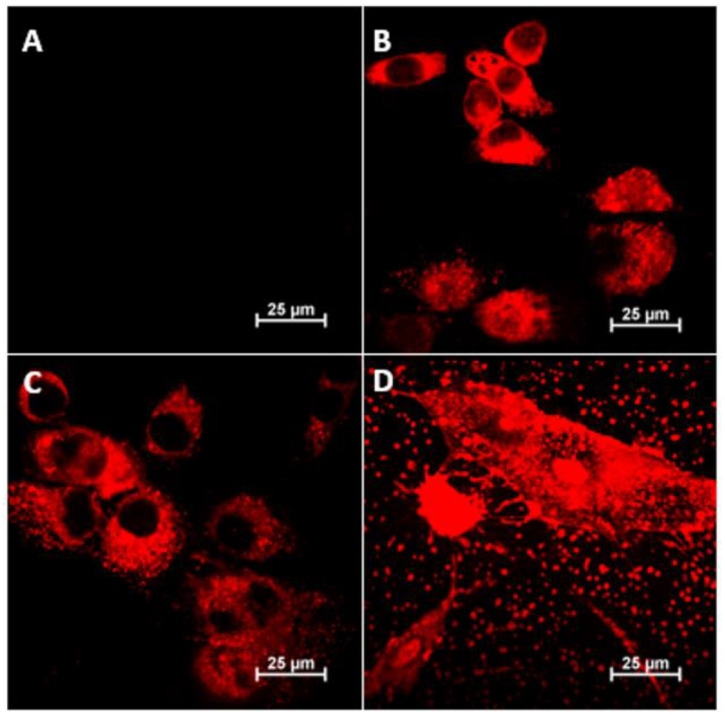
Confocal images, (**A**): control, (**B**): BBGGTMAC1, (**C**): BBGGTMAC2, and (**D**): BBGGTMAC3. 3T3-L1 cell line, polymer concentration 100 μg/mL, exposition time 24 h in 10% serum media, TRITC channel.

The Figure 6 below is a compilation of images taken using confocal microscopy (projections of full 3D images are included in the Supplementary Figure S13) showing the locations of the three polycations (fluorescently labeled) in fibroblast (3T3-L1) cells after 24 h of exposure. The Supplementary Materials also include summaries of the other fluorescence channels and transmitted light images for the images shown below (Figures S9–S12).

Comparing the microscopic images in the figure above, one should postulate that in the case of polycations BBGGTMAC1 and BBGGTMAC2, they localize mainly in the cytoplasm. The situation is quite different in the case of the most cationic polymer, BBGGTMAC3, where the fact of fluorescence of almost the entire cell with no visible gaps may suggest partial accumulation on the cell surface that is, in practice, on a biological membrane. Such conclusions are also confirmed by images recorded for this system at lower concentrations (photos in the Supplementary Materials). At this concentration, the polycation is also bound to residues from disintegrated cells bound to the surface of the slides (there were no such fragments in images for the 50 μg/mL concentration, see Supplementary Figure S10). These fragments can also be associated with fragments of the biological membrane of cells.

**Figure 7:**
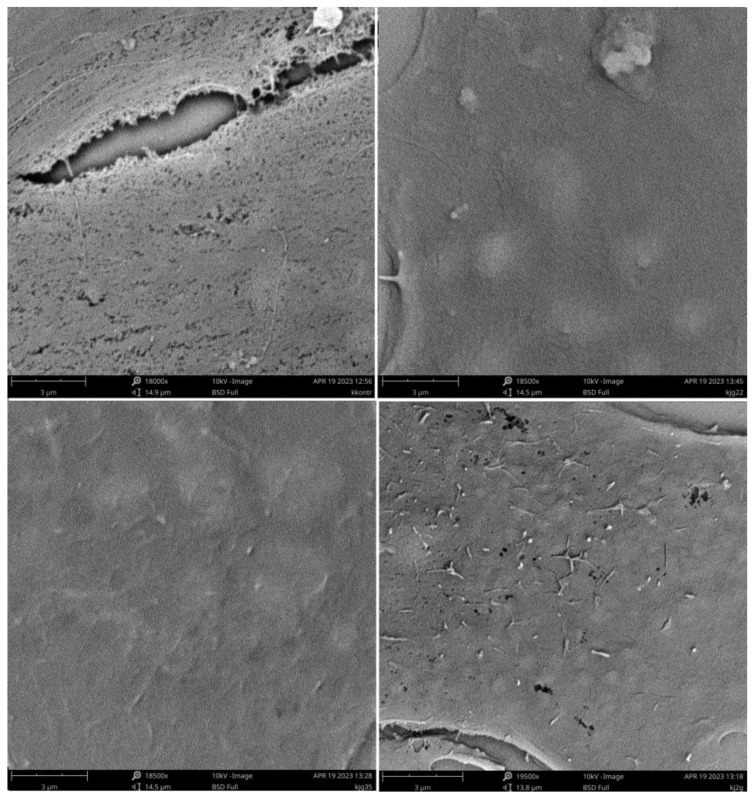
SEM images: top left to right control, BBGGTMAC1, bottom left to right, BBGGTMAC2 and BBGGTMAC3. 3T3-L1 cell line, polymer concentration 100 μg/mL, exposition time 24 h in 10% serum media.

A detailed look at the state of cell surfaces and information on the potential interaction of polymers with biological membranes can be provided by the SEM microscopy, as seen in the images shown below (Figure 7).

The process of fixation and sample preparation for SEM measurements partially removes the lipid layer surrounding the cell which, nevertheless, compares the microscopic images. Characteristic structures are visible and can be identified with the processes taking place within this layer under the influence of polymers. Comparing the samples for the polymers with the control, it can be seen that the presence of polycations prevents the complete removal of the smooth layer from the cell surface. For systems with a greater degree of cationization, such as BBGGTMAC2 and BBGGTMAC3, apart from this smooth layer, we observed additional oblong submicrometer structures occurring in a significant amount for the most cationic polymer. Another additional element found only in the case of polymer BBGGTMAC3 is holes in the surface of the cells that can signal weaker areas of the cell membrane that are prone to rupture. Their formation as a result of the interaction with the lipid layer may be the cause of the toxicity in this polymer.

## 4. Conclusions

The authors have synthesized and characterized, physiochemically, three well-defined cationic derivatives of barley-derived betaglucans obtained in reaction with glycidyltrimethylammonium chloride (BBGGTMAC) with different degrees of cationization (50, 70, and 100% per one glucose unit for, respectively, BBGGTMAC 1, BBGGTMAC2, and BBGGTMAC3) and electrostatic charge. The latter was quantified using zeta potential measurements. The differences in polymer masses were relatively minor, and the two polymers with the highest degrees of modification were practically negligible. Using these polymers, the effect of polycations on cell welfare was investigated by employing the 3T3-L1 and A549 cell lines. It was shown that this biological property strongly depends on the degree of the cationic modification of macromolecules and thus the charge. This relationship does not proceed linearly. Initially, for BBGGTMAC1 and BBGGTMAC2 polymers, we observed a pro-proliferation effect, while at the highest degree of modification and the highest positive charge (highest zeta potential) for BBGGTMAC3, we observed toxicity.

To explain this effect, we studied the interactions of the obtained polymers with the selected models of biological structures. These included BSA and bovine serum proteins, heparin as an example of glycosaminoglycan, DNA, and structures representing biological membranes. Comparing BBGGTMAC polymers with each other, we showed that regardless of charge, they interact in a similar way with heparin and nucleic acids due to differences in electrostatic charges. The biggest differences between the polymers were observed for the interactions with the model bilayers where the polymer with the highest charge, BBGGTMAC3, interacted qualitatively and quantitatively differently than the polymers with lower charges. There is no doubt that the main interactions that polycations undergo in physiological fluids will be polyplex formation ionic-type interactions. This is shown by the results obtained concerning the interaction of BBGBTMAC, regardless of the degree of modification with heparin and nucleic acids. This is a feature that will strongly affect the activity of polycations but, as our studies show, is not necessarily responsible for their toxicity in mammalian cells. It should also be noted that the phenomena of ionic interactions between charged macromolecules will, in practice, strongly depend on the intermediate environment of these polymers [34], which under biological conditions can vary greatly.

Images obtained using confocal microscopy show a difference in the accumulation of polycations depending on the degree of cationic modification, indicating that BBGGTMAC3 interacts with the cell membrane, and BBGTMAC1 and BBGTMAC1 have only penetrated the cytoplasm. This can be attributed to changes in the bilayer organization of the cell membrane, such as changes in the electrostatic field gradient between membrane layers and the general structure of the cell membrane. e.g., its thickness and the orientation of acyl chains [35].

Regardless of membrane effects, data obtained for polymer serum mixture-based models using dynamic light scattering may indicate a significant interaction of polycations with non-albumin proteins. It can be assumed that we are dealing with an interaction with growth factors, which would explain the partial pro-proliferative effects in cultures with serum. This may partially account for the change in cell welfare modeling by polycations but will be limited mainly to cell cultures in a medium with serum, while the characteristic effect is also observed significantly in cultures without serum.

Proposing the mechanism responsible for the toxicity of polycations and the characteristic chemical properties that cause it is critical to the development of polymeric drugs based on such macromolecules. The ability of polycations to react with such important biomacromolecules as nucleic acids and glycosaminoglycans provides the basis for modifying their biological activity. This suggests that polycations, as long as they are not toxic, should have broad potential to model the biological activity of other macromolecules in living organisms. And here, one should recall studies on the neutralization of heparin or the use of polycations for gene therapy as vectors.

## Figures and Tables

**Table 1:** The exact amounts of NaOH, GTMAC, and water used in the cationization process.

Name	NaOH (mg)	GTMAC (mL)	Water (mL)
BBGGTMAC1	200	6	50
BBGGTMAC2	200	12	50
BBGGTMAC3	200	12 + 6	50

**Table 2:** Number, average weight, molecular weight (Mn and Mw), molecular weight distribution PDI (M_w_/M_n_), and degree of polymerization (DP) of the obtained polymers based on elemental analysis (EA) and ^1^H NMR.

Name	DP (n) EA	DP (n) ^1^H NMR	Zeta Potential [mV]	M_n_∙10^4^ [Da]	M_w_∙10^4^ [Da]	PDI	LogP *
BBGGTMAC1	57	50	29.53 ± 0.74	1.50	1.77	1.19	−5.71
BBGGTMAC2	83	71	43.33 ± 0.94	8.57	11.0	1.28	−7.43
BBGGTMAC3	100	100	46.67 ± 0.25	12.6	14.9	1.18	−7.85

* LogP was determined with the site molinstpiration.com and was conducted for a disaccharide based on a simplified betaglucan structure with one mer to which cationic groups were attached (BBGGTMAC1), trisaccharide with two mers (BBGGTMAC2), or three mers (BBGGTMAC3) with the cationic groups.

## Data Availability

Data is contained within the article or supplementary material.

## Supplementary Materials

The following supporting information can be downloaded at https://www.mdpi.com/article/10.3390/pharmaceutics15072009/s1, Table S1: Combustion elemental analysis of the obtained polymers; Figure S1: IR spectra of the obtained polycations; Figure S2: Dependence of unbound % heparin on the polymer in the solution; Figure S3: H1NMR spectrum of BBGGTMAC1, BBGGTMAC2, and BBGGTMAC3 in D2O. Figure S4: Calorimetric titrations of 200 µM polycation solutions into a 20 µM BSA solution after subtraction of the dilution heats. Experiments were performed in PBS at 37 °C; Figure S5: Calorimetric titrations of 200 µM polycation solutions into a 20 µM BSA solution after subtracting the dilution heats. Experiments were performed in PBS at 37 °C; Table S2: Thermodynamic parameters obtained when analyzing the calorimetric data of the interaction of the polycations with DNA based on a single class of a binding sites model. Figure S6: Particle size distribution of the polycations; Figure S7: The diameters of the objects formed based on dynamic light scattering as a result of the interaction of the polymers with DNA. From left to right: BBGGTMAC1, BBGGTMAC2, BBGGTMAC3; Figure S8: Example calorimetric titrations of the 5 mM DNA solutions into a 500 µM polymer solution. Experiments were performed in PBS at 37 °C. Solid lines represent the best fit of the single-site binding model to the data; Figures S9–S13: Confocal cell images; Table S3: Depolymerization process analysis.
