# Supplementary material for: The Use of a Barley-Based Well to Define Cationic Betaglucan to Study Mammalian Cell Toxicity Associated with Interactions with Biological Structures
Source: Pharmaceutics. 2023 Jul 23;15(7):2009. doi: 10.3390/pharmaceutics15072009 (PMC10385077; doi:10.3390/pharmaceutics15072009)
Supplement: Supplementary file 1 [file pharmaceutics-15-02009-s001.zip › pharmaceutics-2493688-supplementary.pdf]

pharmaceutics

Supplement

The Use of a Barley-Based Well to Define Cationic Betaglucan to StudyMammalian Cell Toxicity Associated with Interactions with Biological Structures

Malgorzata Tymecka [1,2], Katarzyna Hac-Wydro [2], Magdalena Obloza [2] and Piotr Bonarek [3] and Kamil Kaminski [2,*]

[1] Doctoral School of Exact and Natural Sciences, Faculty of Chemistry, Jagiellonian University, Gronostajowa 2, 30-387 Kraków, Poland; m.tymecka@doctoral.uj.edu.pl
[2] Faculty of Chemistry, Jagiellonian University, Gronostajowa 2, 30-387 Kraków, Poland; katarzyna.hac-wydro@uj.edu.pl (K.H.-W.); m.obloza@uj.edu.pl (M.O.)
[3] Department of Physical Biochemistry, Faculty of Biochemistry, Biophysics and Biotechnology, Jagiellonian University, Gronostajowa 7, 30-387 Krakow, Poland; piotr.bonarek@uj.edu.pl
* Correspondence: kaminski@chemia.uj.edu.pl

Table S1. Combustion elemental analysis of obtained polymers.

Name	Elemental composition %		
	N	C	H
**Betaglucan**	1.77 ± 0.07	40.82 ± 0.58	6.19 ± 0.03
**BBGGTMAC3**	4.44 ± 0.02	45.15 ± 0.01	7.52 ± 0.04
**BBGGTMAC2**	3.59 ± 0.03	40.57 ± 0.02	7.55 ± 0.12
**BBGGTMAC1**	2.92 ± 0.04	41.41 ± 0.21	7.18 ± 0.06

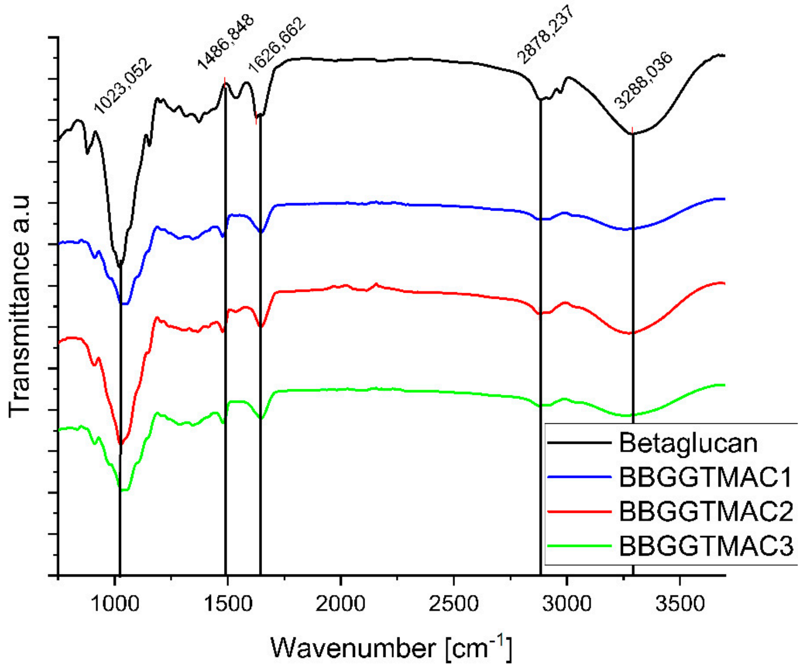

Wavenumber [cm^{-1}]	Functional groups
1023,052	-CO stretching
1486,848	-CH_3 from cationic group
1626,662	-NH bending
2878,237	-CH stretching
3288,036	-OH stretching

Figure S1. IR spectra of the obtained polycations with marked peaks and functional groups

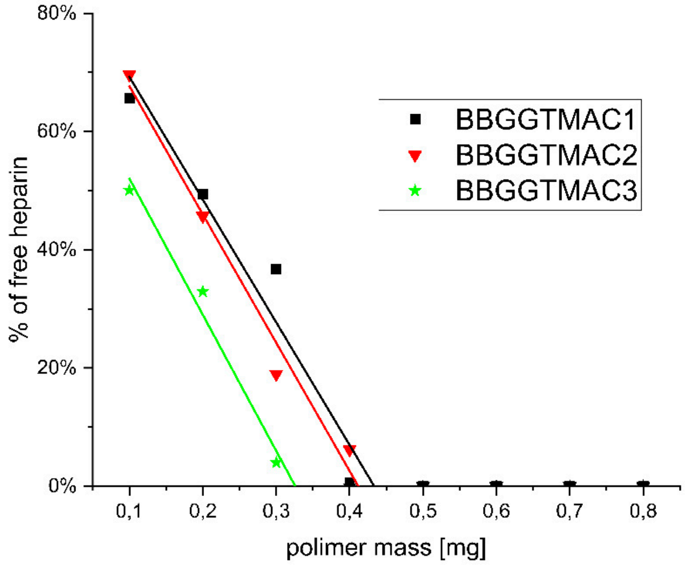

Figure S2. Dependence of unbound % heparin on polymer in solution

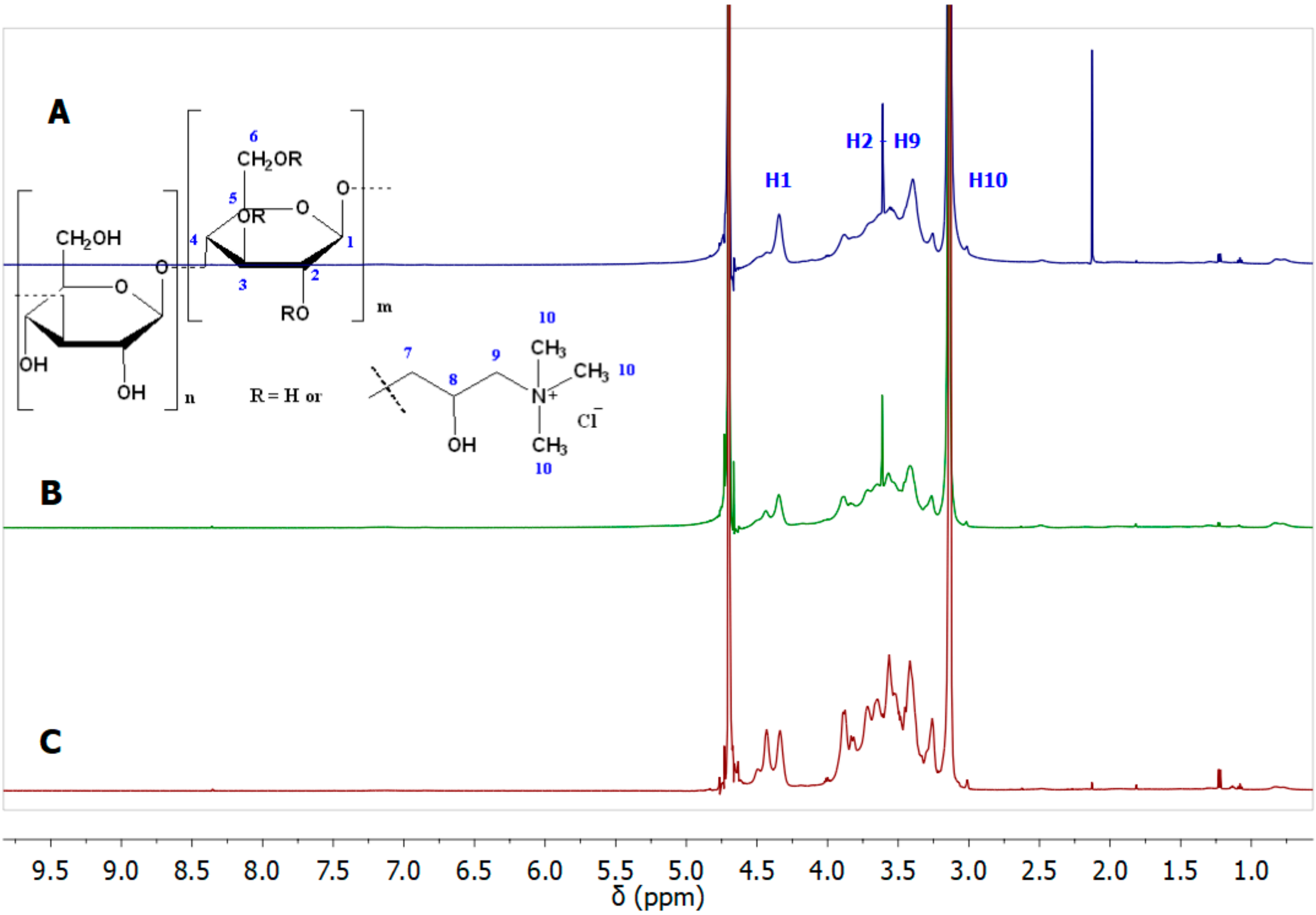

Figure S3. H[1]NMR spectrum of BBGGTMAC1, BBGGTMAC2 and BBGGTMAC3 in D_2O.

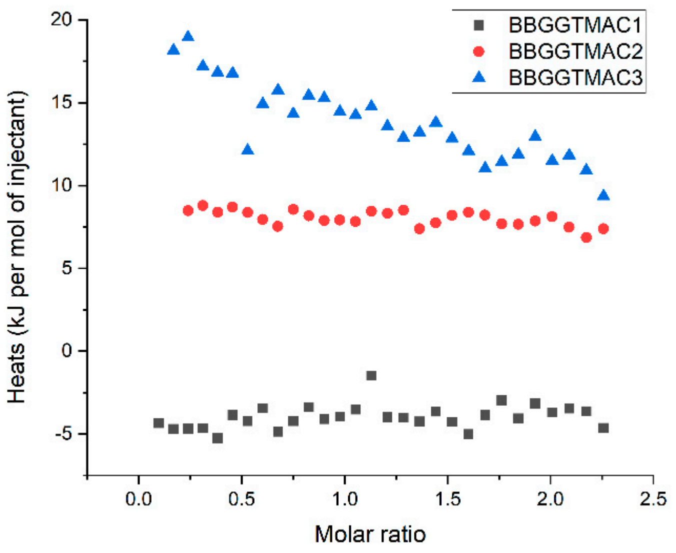

Figure S4. Calorimetric titrations of 200 μM polycations solutions into 20 μM BSA solution after subtraction of the dilution heats. Experiments were performed in PBS at 37°C.

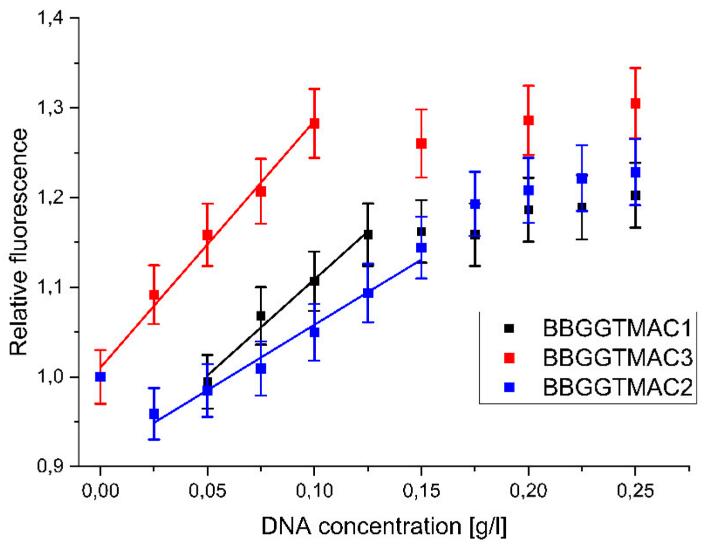

Figure S5. Change in fluorescence spectra of polycations with addition of DNA (polycation concentration 0,05 g/l).

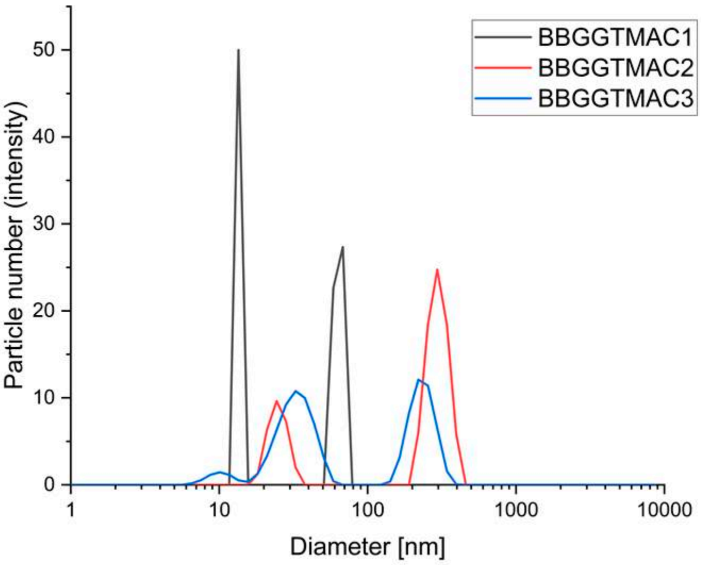

Figure S6. Particle size distribution of polycations.

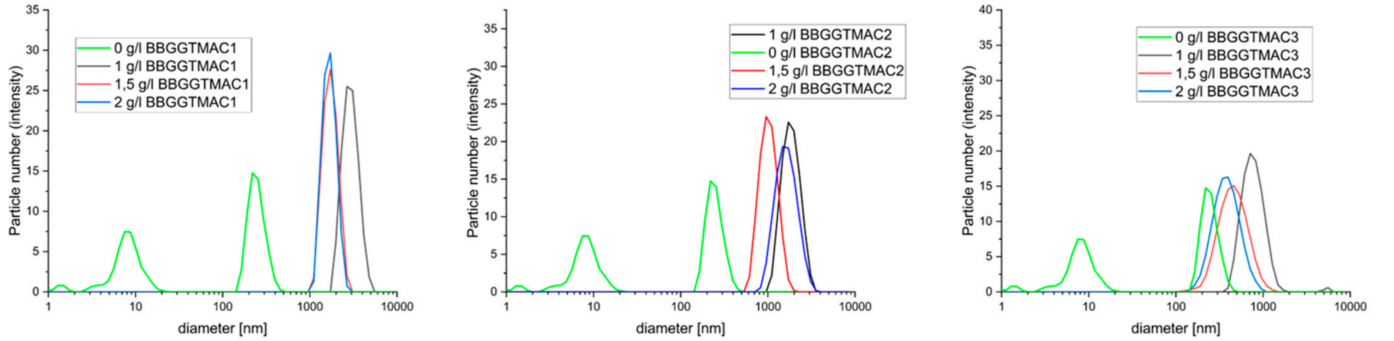

Figure S7. The diameters of the objects formed based on dynamic light scattering in result of the interaction of polymers with DNA, from left to right BBGGTMAC1, BBGGTMAC2, BBGGTMAC3

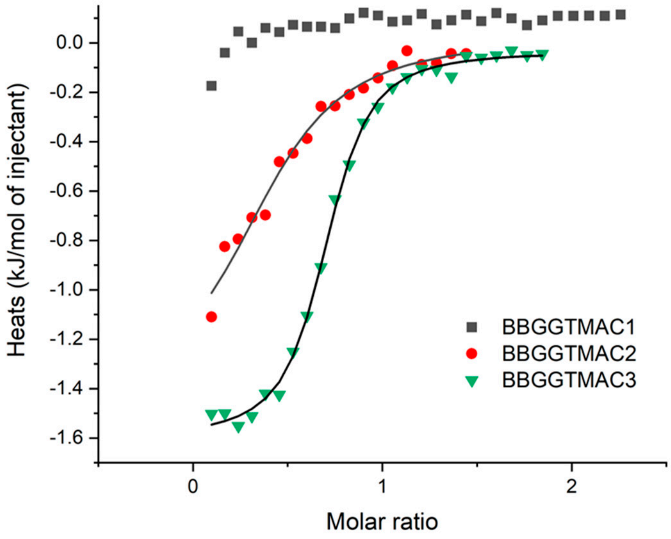

Figure S8. Example calorimetric titrations of 5 mM DNA solutions into 500 μM polymers solution. Experiments were performed in PBS at 37°C. Solid lines represent the best fit of the single-site binding model to the data.

Table S2. Thermodynamic parameters obtained when analyzing calorimetric data of the interaction of polycations with DNA based on a single class of binding sites model.

Name	stoichiometry	K_{app} [×10^3 M^{-1}]	ΔH_{app} [kJ/mol]	ΔS_{app} [J/mol/K]
BBGGTMAC2	0.46 ± 0.05	14 ± 6	-1.5 ± 0.3	18 ± 1
BBGGTMAC3	0.66 ± 0.05	97 ± 13	-2.0 ± 0.1	21 ± 0.3

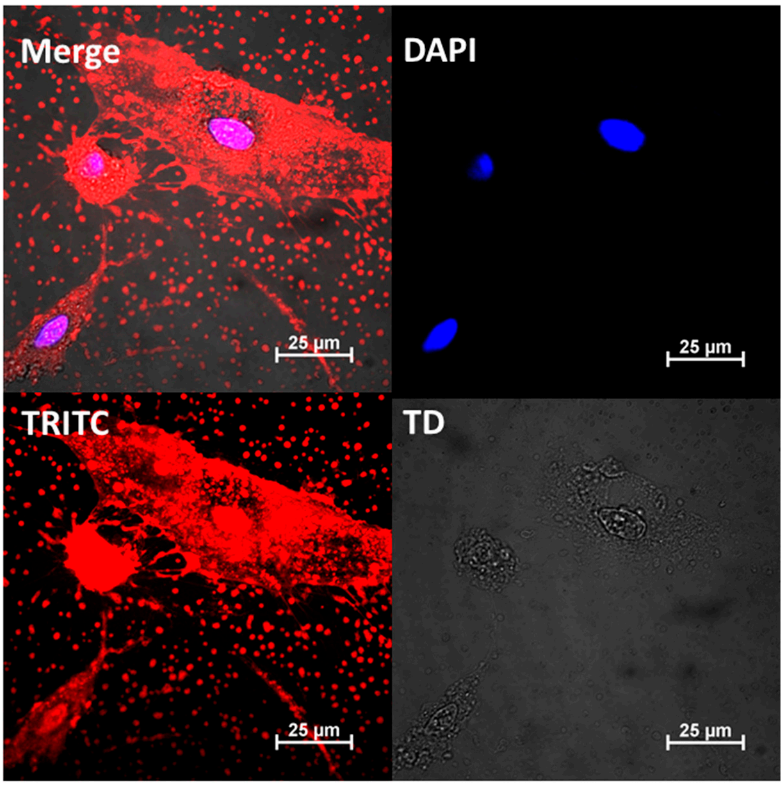

Figure S9. Confocal images of BBGGTMAC3.

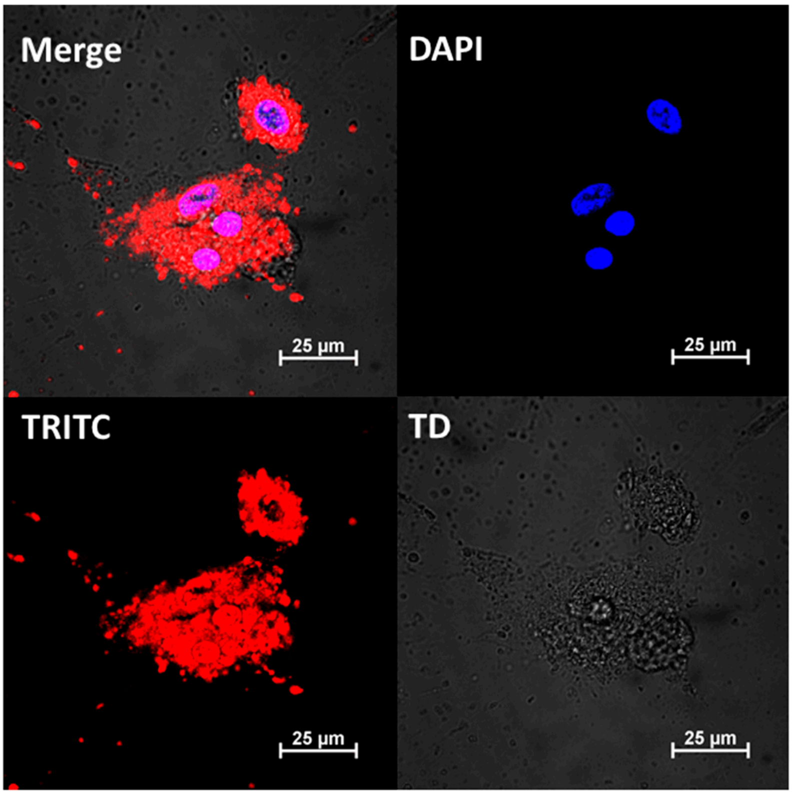

Figure S10. Confocal images of BBGGTMAC3 (lower concentration 50ug/ml).

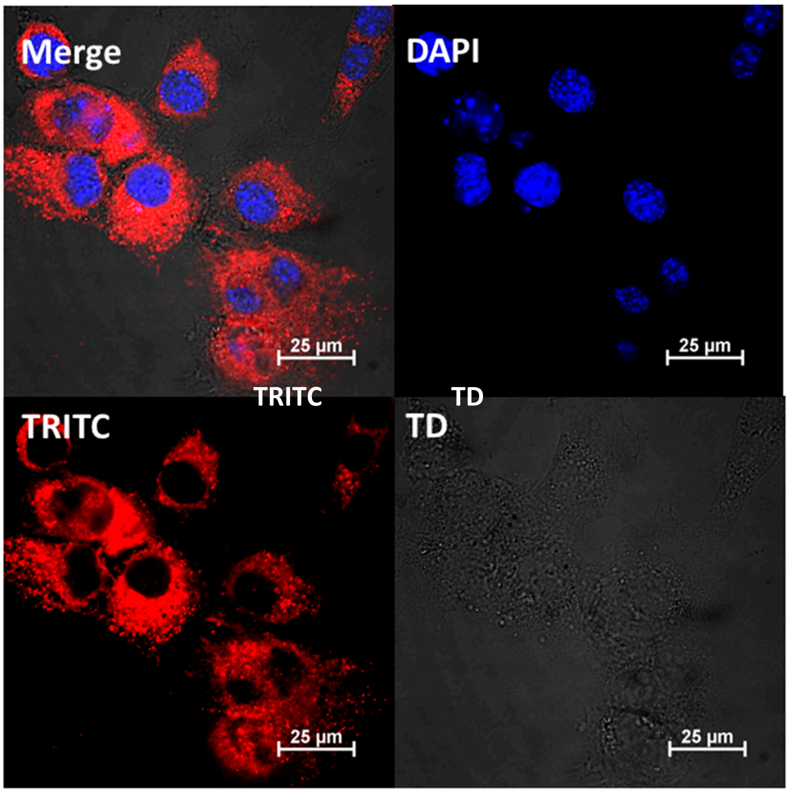

Figure S11. Confocal images of BBGGTMAC1.

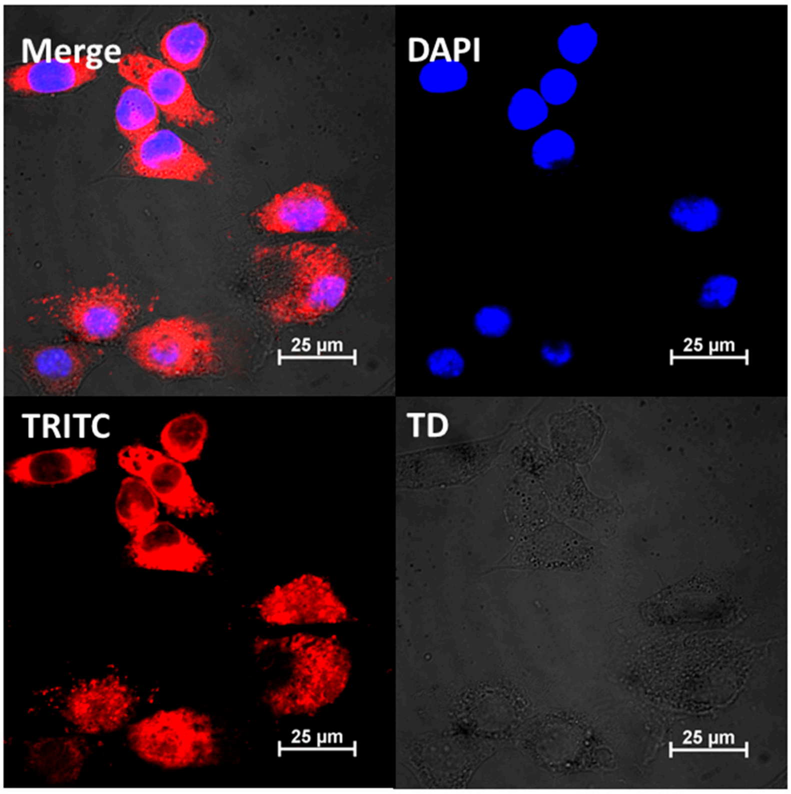

Figure S12. Confocal images of BBGGTMAC2

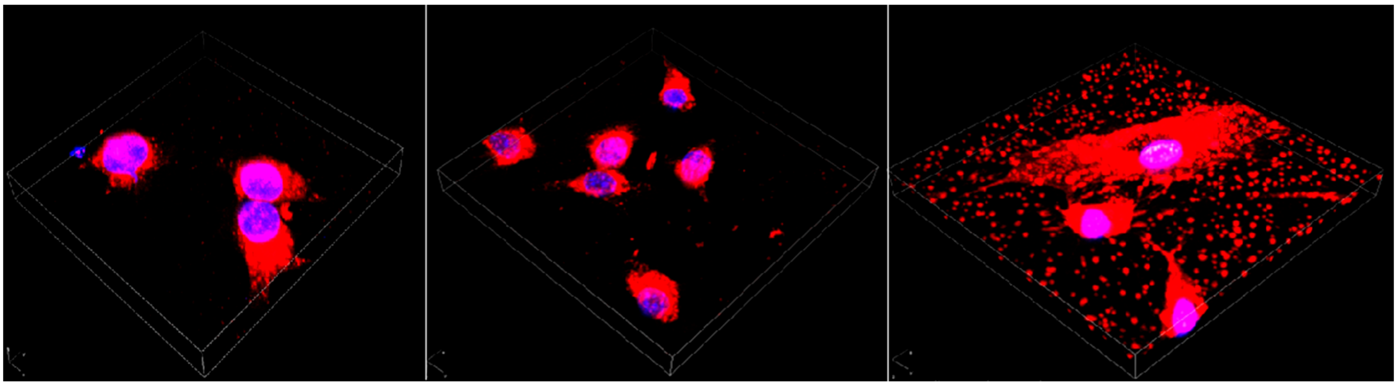

Figure S13. 3D Confocal images of left to right: BBGGTMAC1, BBGGTMAC2, BBGGTMAC3.

Table S3. Depolymerization process analysis.

Name	Time passed after the addition of betaglucanse [min]	Flow time [s]
**Water**	-	92
**BBGGTMAC2**	-	141
**BBGGTMAC2 + 5 mg betaglucanase**	0	134
	15	130
	45	130
**BBGGTMAC2 + 25 mg betaglucanase**	0	125
	15	124
	30	123
	45	123
